# Nested association mapping-based GWAS for grain yield and related traits in wheat grown under diverse Australian environments

**DOI:** 10.1007/s00122-022-04230-9

**Published:** 2022-10-07

**Authors:** Charity Chidzanga, Daniel Mullan, Stuart Roy, Ute Baumann, Melissa Garcia

**Affiliations:** 1 School of Agriculture, Food and Wine, The University of Adelaide, Waite Campus, PMB 1 Glen Osmond, Adelaide, SA 5064 Australia; 2 ARC Industrial Transformation Research Hub for Wheat in a Hot and Dry Climate, Waite Research Institute, The University of Adelaide, Glen Osmond, SA 5064 Australia; 3 InterGrain Pty Ltd, 19 Ambitious Link, Bibra Lake, WA 6163 Australia; 4 Present Address: Inari Agriculture, One Kendall Square, Building 600/700, Suite 7-501, Cambridge, MA 02139 USA

## Abstract

**Key message:**

Utilising a nested association mapping (NAM) population-based GWAS, 98 stable marker-trait associations with 127 alleles unique to the exotic parents were detected for grain yield and related traits in wheat.

**Abstract:**

Grain yield, thousand-grain weight, screenings and hectolitre weight are important wheat yield traits. An understanding of their genetic basis is crucial for improving grain yield in breeding programmes. Nested association mapping (NAM) populations are useful resources for the dissection of the genetic basis of complex traits such as grain yield and related traits in wheat. Coupled with phenotypic data collected from multiple environments, NAM populations have the power to detect quantitative trait loci and their multiple alleles, providing germplasm that can be incorporated into breeding programmes. In this study, we evaluated a large-scale wheat NAM population with two recurrent parents in unbalanced trials in nine diverse Australian field environments over three years. By applying a single-stage factor analytical linear mixed model (FALMM) to the NAM multi-environment trials (MET) data and conducting a genome-wide association study (GWAS), we detected 98 stable marker-trait associations (MTAs) with their multiple alleles. 74 MTAs had 127 alleles that were derived from the exotic parents and were absent in either of the two recurrent parents. The exotic alleles had favourable effects on 46 MTAs of the 74 MTAs, for grain yield, thousand-grain weight, screenings and hectolitre weight. Two NAM RILs with consistently high yield in multiple environments were also identified, highlighting the potential of the NAM population in supporting plant breeding through provision of germplasm that can be readily incorporated into breeding programmes. The identified beneficial exotic alleles introgressed into the NAM population provide potential target alleles for the genetic improvement of wheat and further studies aimed at pinpointing the underlying genes.

**Supplementary Information:**

The online version contains supplementary material available at 10.1007/s00122-022-04230-9.

## Introduction

Grain yield (GY) determines the efficiency of wheat production and food security. In wheat breeding, the main focus is to increase GY. Being the result of many processes occurring within the plant and their interaction with the environment, GY is directly and indirectly influenced by other traits. Often traits that directly and indirectly influence GY are used in wheat breeding to improve GY. In Australia, there are three traits which are key targets for wheat breeding: thousand-grain weight (TGW), screenings (SCG), and hectolitre weight (HW). TGW, defined as the weight of one thousand seeds selected at random, is an essential trait that directly influences GY increase under both favourable and stressful environments (Kuchel et al. 2007). SCG are the proportion of wheat grains that falls through a 2-mm slotted screen after a defined number of shakes/agitations. SCG are negatively correlated with GY and can be used as a yield-related trait, especially under stressful conditions when seeds can be smaller. Some genotypes produce more SCG under stressful conditions than others. In Australia, SCG are an important trait because they greatly determine the commercial value and flour yield of wheat. HW is another crucial trait in determining the commercial value of wheat. Being the weight of 100 L, it is an indicator of grain quality (cleanness, plumpness and packing density) and flour yield. Understanding the genetic basis of TGW, SCG and HW together with GY are critical for improving GY in breeding programmes (Wu et al. 2012).

GY and related traits are controlled by many genes and highly influenced by the environment. The quantitative nature of these traits coupled with the limited knowledge of their genetic architecture presents a challenge to their improvement through breeding. In an endeavour to understand the genetic basis of these traits and improving their trait values through breeding, linkage analysis in bi-parental mapping populations (Kuchel et al. 2007; Li et al. 2021; Maphosa et al. 2014) and genome-wide association studies (GWAS) in diversity panels (Garcia et al. 2019; Schmidt et al. 2020) have been employed. Though successful in identifying quantitative trait loci (QTL), each of these mapping populations has their limitations. Bi-parental populations lack allelic diversity and have low resolution (Yu et al. 2008). Diversity panels have allelic diversity and high resolution but are limited by the confounding effects of population structure (Zhang et al. 2010). Nested association mapping (NAM) populations are valuable genetic resources that combine the strengths of bi-parental populations and diversity panels (Myles et al. 2009; Poland et al. 2011). NAM populations have the advantages of high allelic diversity, high mapping resolution and low sensitivity to population structure (Yu et al. 2008). In addition to enabling mapping of QTL, NAM populations also complement conventional breeding approaches by increasing genetic diversity and providing useful germplasm to breeding programmes (Scott et al. 2020). In the development of NAM populations, a diverse set of founder lines, typically more than ten, are crossed to one or more well-characterised and/or locally adapted elite line(s) (Chidzanga et al. 2021; Fragoso et al. 2017; Yu et al. 2008). The resultant F_1_ goes through at least four generations of selfing to produce recombinant inbred lines (RILs) whose genomes are mosaics of the parental genomes (Yu et al. 2008). Shuffling of the parental genomes breaks down population structure, introduces recent recombinations and creates new allele combinations (McMullen et al. 2009). This aids in detecting small effect QTL and rare alleles from specific parents (McMullen et al. 2009).

When coupled with phenotypic evaluations in diverse environments, NAM populations present a more powerful approach for detecting QTL. However, NAM populations tend to be large, with as many as 6280 lines being reported (Kidane et al. 2019) and are therefore difficult to evaluate at one time due to constraints in space, time, labour and funds. As a result, NAM populations are evaluated in unbalanced (not all genotypes in all environments), multi-environment trials (MET). Though it is common practice in plant breeding to evaluate genotypes in METs, the unbalanced nature of the trials and the presence of genotype by environment interactions (GEI) pose a challenge in the analysis of MET data (Smith and Cullis 2018) and consequently QTL mapping. Considering the advantages presented by NAM multi-environment QTL mapping, it is crucial to have a genetic and statistical model that appropriately models the genetic variance across environments and genetic covariance between pairs of environments to increase the power to detect QTL. One such model is the one-stage factor analytic linear mixed model (FALMM) (Beeck et al. 2010; Gogel et al. 2018; Smith and Cullis 2018). The FALMM accounts for the covariances of the *G* × *E* effects between environments using unknown common factors, which are estimated from the data. Pedigree information (Oakey et al. 2006, 2007) can also be included into the model enabling the portioning of *G* × *E *effects into additive and non-additive *G* × *E* effects, and their respective between environment genetic variances matrices can be modelled with separate FA models (Oakey et al. 2007; Smith and Cullis 2018). The model also accommodates unbalanced data and the individual trial designs to accurately explore and exploit GEI (Beeck et al. 2010). Applying the FALMM to NAM MET data fully exploits the benefits of the NAM in QTL mapping.

In the present study, we aimed to understand the genetic basis of GY, TGW, SCG and HW in the large-scale OzNAM wheat population (Chidzanga et al. 2021) that was grown and evaluated under diverse Australian conditions for three years, by applying a single-stage FALMM to NAM MET data and then conducting a genome-wide association study (GWAS).

## Materials and methods

### Plant material and experimental design

In our previous study, we describe the development of the OzNAM population by crossing and backcrossing 73 diverse exotic parents (selected for diversity in terminal drought and heat stress, nitrogen use efficiency and originating from countries with dry and hot weather conditions) to two Australian elite varieties Gladius and Scout (Chidzanga et al. 2021). We also demonstrated the utility of the population in QTL mapping by mapping QTL for maturity and plant height using a subset of the population consisting of 530 lines (Chidzanga et al. 2021). In this study, we used a total of 2530 RILs from 124 NAM families, derived from the OzNAM population (Chidzanga et al. 2021) and twenty-eight check varieties (Supplementary Table 1) to evaluate in multi-environment trials (MET) and map marker-trait associations for GY, TGW, SCG and HW. For each NAM family, there were 8–51 RILs. Due to the size of the NAM population and the challenges of evaluating the entire population at once in a single trial, the population was split into three sets of varying sizes. Each set was evaluated under field conditions in a single environment in the first year before a subset of each set was selected for further evaluation in at least two sites in subsequent years. Selection of the subsets was carried out as described in Chidzanga et al. (2021). Overall, there were 12 trials located in South Australia, Western Australia and New South Wales during the years from 2017 to 2020. Details of the NAM METs are given in Table 1. Trials 17RSW-NAM_A, 18RSW-NAM_A, 18DND-NAM_A and 19RSW-NAM_A were reported in our previous study with regard to maturity and plant height data (Chidzanga et al., 2021). In the current study, we report on new traits measured from previously reported trials and new trials. Therefore, all the phenotypic data reported in this study are new.

**Table 1:** Number of NAM RILs from each of the three sets of the OzNAM population and the calendar years, multi-environment trial (MET) years, geographic locations and sowing dates for each of the twelve trials

Set	Number of RILs	Year		Evaluation Site	State	Latitude	Longitude	Sowing date	Trial name
		Calendar	MET						
A	530	2017	1	Roseworthy	SA	− 34.521957	138.662206	18/05/2017	17RSW-NAM_A
	238	2018	2	Roseworthy	SA	− 34.515090	138.668496	18/05/2018	18RSW-NAM_A
	238	2018	2	Dandaragan	WA	− 30.685308	115.665099	07/06/2018	18DND-NAM_A
	238	2019	3	Roseworthy	SA	− 34.514273	138.689983	18/05/2018	19RSW-NAM_A
	238	2019	3	Lockhart	NSW	− 35.07866	146.77468	30/05/2019	19LKH-NAM_A
B	1268	2018	1	Roseworthy	SA	− 34.484985	138.682256	02/07/2018	18RSW-NAM_B
	537	2019	2	Roseworthy	SA	− 34.514273	138.689983	18/05/2019	19RSW-NAM_B
	539	2019	2	Dandaragan	WA	− 30.685308	115.665099	25/05/2019	19DND-NAM_B
	229	2020	3	Moora	WA	− 30.63072	116.07587	21/05/2020	20MRA-NAM_B
C	376	2019	1	Roseworthy	SA	− 34.514273	138.689983	23/05/2019	19RSW-NAM_C
	366	2019	1	Dandaragan	WA	− 30.685308	115.665099	25/05/2019	19DND-NAM_C
	229	2020	2	Moora	WA	− 30.63072	116.07587	21/05/2020	20MRA-NAM_C

Due to the size of the NAM population and the challenges of evaluating the entire population at once in a single trial, the population was split into three sets of varying sizes (A, B, C). Each set was evaluated under field conditions in a single environment in the first year before a subset of each set was selected for further evaluation in at least two sites in subsequent years (MET years)

Trials were rainfed and managed following local practices. Environmental data (e.g. temperature and rainfall) were collected from weather stations less than 5 km from each trial site (Supplementary Table 2). In this study, a trial was defined as a combination of year, site and NAM set evaluated and is synonymous with environment. Each trial was sown in a row-by-range array and was designed as a partially replicated experimental design (Cullis et al. 2006). In total, there were 8688 plots across the 4 years. Some check varieties had additional replication of up to ten plots in a single trial. Trials were unbalanced, and check varieties were used to improve connectivity of the genotypes across trials. Six of the twenty-eight check varieties were present in all trials (Supplementary Table 1). To further improve the concurrence of varieties between trials, trials that were grown adjacent to each other in a particular site were combined during analysis. As a result, the number of trials was reduced from twelve to nine. Table 2 lists all the trials and their new names. The overall connectivity between trials differed with traits as some traits was not measured in all trials.

**Table 2:** List of the nine NAM multi-environment trial (MET) Environments defined by a combination of their associated year, site and NAM set

Year	Site	NAM set	Year*Site*NAM set (environment)
2017	Roseworthy	Set A	17RSW-NAM_A
2018	Dandaragan	Set A	18DND-NAM_A
2018	Roseworthy	Set A	18RSW-NAM_A
2018	Roseworthy	Set B	18RSW-NAM_B
2019	Dandaragan	Set B	19DND-NAM_B
2019	Dandaragan	Set C	19DND-NAM_C
2019	Lockhart	Set A	19LKH-NAM_A
2019	Roseworthy	Sets A, B and C	19RSW-NAM_ABC
2020	Moora	Sets A and B	20MRA-NAM_BC

### 2.2 Phenotyping

Phenotypic data were collected from plots for GY, TGW, SCG and HW. GY was measured as the mass of the harvested grain per plot converted to tonnes per hectare, TGW (g) was measured as the weight of a sample of one thousand grains, and SCG was measured by collecting and weighing the proportion of material (including wheat grains and chaff) from a test sample that fell through a 2-mm sieve after 40 shakes. SCG was expressed as a percentage of the sample weight. HW was measured by weighing the grain collected from a levelled 500 ml measuring container of a chondrometer (Graintec Scientific Pty Ltd, Australia) and converting the weight to kilograms per hectolitre (kg/HL). In the 19RSW-NAM_ABC trial, TGW was not measured for the B and C sets and so the TGW of the 19RSW-NAM_ABC trial is comprised of NAM set A only. HW was not measured in the 18RSW-NAM_B trial due to the trial being harvested late and therefore being deemed as unrepresentative.

### Statistical analysis

Phenotypic data from all the trials were analysed in R using the R package ASReml-R version 4.1. Individual raw plot data across all the trials were combined and analysed in a one-stage factor analytic linear mixed model analysis (FALMM) with pedigree information (Smith et al. 2001; Oakey et al. 2007; Kelly et al. 2007; Gogel et al. 2018; Smith et al. 2021). The pedigree information added to the model enabled the *G* × *E* effects to be portioned into additive and non-additive effects. The mathematics, genetic variance and covariance structures of the model including pedigree information are described in Beeck et al. (2010) and Gogel et al. (2018). In general, the residual effects of each trial were modelled by including terms that account for the randomisation processes used in the trial design and using spatial methods to account for plot-to-plot variation (Gilmour et al. 1997; Stefanova et al. 2009). Once the spatial structures for each trial were appropriately modelled and outliers removed, a factor analytic linear mixed model of order 2 (FA2 model) (Smith et al. 2001) was used to model the genotype by environment effects. The factors of the additive and non-additive FA model were increased until at least 80% of the total additive variance was accounted for. The analysis generated genetic correlations between pairs of trials and these correlations were used as a measure of the *G* × *E* interactions (GEI). Outliers were detected using standardised conditional residuals and were removed to reduce bias of estimates. Because the trials were unbalanced, best linear unbiased predictions (BLUPs) (Robinson 1991) of the random genotype effects from the FA2 model were used to predict trait values for genotypes that were not present in any given trial. BLUPs for GY, TGW, SCG and HW from the FA2 model were used to perform the GWAS.

### Genotyping and construction of multi-allelic single-nucleotide polymorphism linkage disequilibrium (SNPLDB) markers

In our previous study, we describe the genotyping of the NAM population using a targeted genotype by sequencing approach to produce both SNP markers and multi-allelic haplotype markers (Chidzanga et al. 2021). Here, we make use of the tGBS SNP markers of Chidzanga et al. (2021) to generate multi-allelic SNP linkage disequilibrium (SNPLDB) markers using the SNPLDB function of the RTM-GWAS programme as described in He et al. (2017). Before the SNPLDB markers were generated, the 16,439 tGBS SNPs were filtered to remove dominant markers, markers called in < 80% of the samples and markers with heterozygosity > 6% resulting in 11,277 SNP markers. To cater for the two recurrent parent structure of the OzNAM, SNPLD programme was customised following three steps. First, genomic blocks were defined based on all NAM RILs. Second, unique haplotypes of a block were determined and numbered based on all parental lines. Finally, the haplotype of the NAM RILs was mapped to parental haplotype according to the pedigree. If the haplotype of inbred line did not match its parental haplotypes, then it was mapped to the most similar parental haplotypes (Jianbo He, National Centre for Soybean Improvement, Nanjing Agricultural University, personal communication). The resulting 5419 SNPLDB markers were then used to construct a genetic similarity matrix and map QTL.

### Nested association mapping-based GWAS

A genetic similarity coefficient (GSC) matrix for estimation and correction of population structure was constructed from the SNPLDB markers as described in He et al. (2017). The top 10 eigenvectors with the largest eigenvalues of the GSC matrix were used as covariates for the correction of population structure in the GWAS. The restricted two-stage multi-locus multi-allele GWAS (RTM-GWAS) procedure (He et al. 2017) was used to map marker-trait associations in 1466, 1863 and 2066 NAM RILs for HW, TGW and SCG and GY, respectively. A significance level of *p* ≤ 0.001 was used for the two stages of RTM-GWAS described by He et al. (2017). The markers that were detected by GWAS as significantly associated with the respective traits were reported as marker-trait associations (MTAs) in this study.

## Results

### Phenotypic variation

**Fig. 1:**
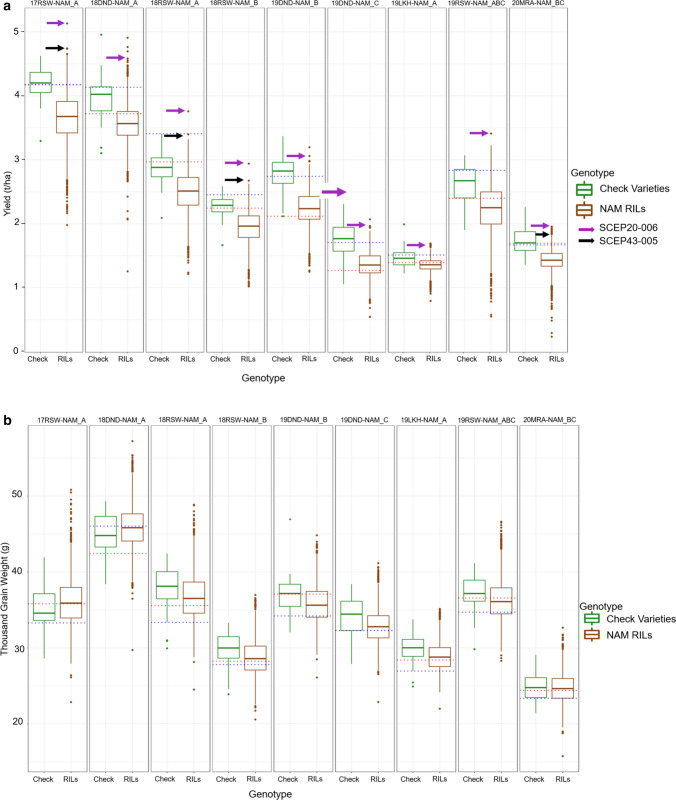

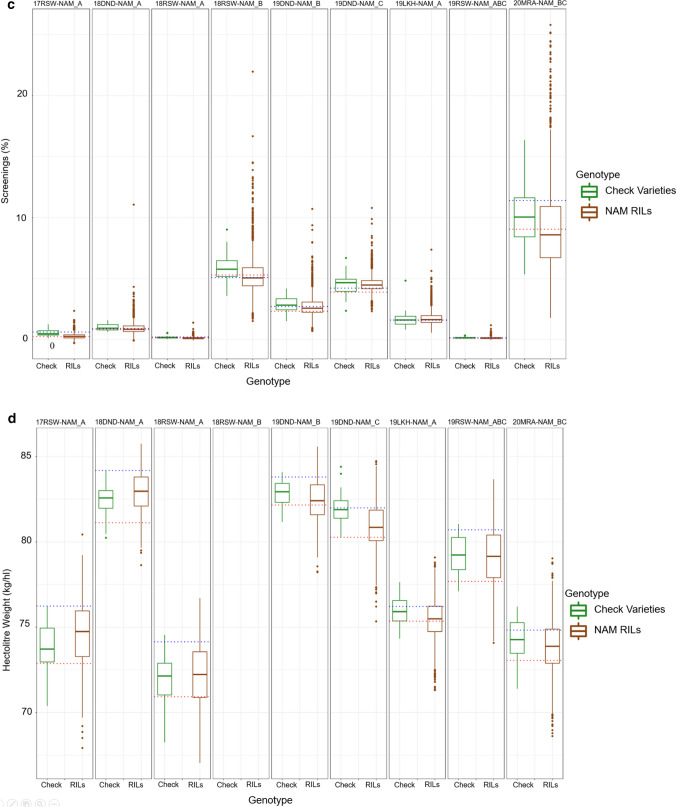
Boxplots showing the distribution of phenotypic BLUPs for the NAM RILs and check varieties for **a** GY **b** TGW **c** SCG **d** HW in nine environments. Boxplots for check varieties are shown in green, and boxplots for the NAM RILs are shown in brown. The red and blue dotted lines indicate the BLUP for the Gladius and Scout recurrent parents, respectively, Gladius and Scout had the same TGW in 19DND-NAM_C. For the GY boxplots, the purple and black arrows indicate SCEP20-006 and SCEP43-005, respectively. Hectolitre weight was not measured in 18RSW-NAM_B

The BLUPs for GY, TGW, SCG and HW ranged from 0.2 to 5.1 t/ha, 15.8 to 57.2 g, 0 to 25.8%, 67.0 to 85.8 kg/hl, respectively, across all the environments. Figure 1 shows the distribution of the BLUPs for the four traits in the NAM RILs in comparison with the check varieties in different environments. The performance of some the NAM RILs was comparable to the performance of the check varieties and the recurrent parents for all the traits. Two NAM RILs (SCEP20-006 and SCEP43-005) had consistently higher GY than most of the check varieties and both the recurrent parents in at least four environments. SCEP20-006 had a consistently higher predicted GY in all of the nine environments, while SCEP43-005 had high GY in four of the environments (Fig. 1a). Supplementary Table 3 records the allele effects and allelic combinations at all significant yield MTA for SCEP20-006 and SCEP43-005. The TGW for the two-high yielding RILs was above average in each environment. Phenotypic correlations among the four traits are shown in Fig. S1. TGW had moderate positive correlations with GY and HW, while SCG was negatively correlated with all the other three traits. There was a weak positive correlation between GY and HW.

The performance of the NAM RILs and the check varieties differed with environment. For GY, TGW, SCG and HW, the environments in which the genotypes performed better were 17RSW-NAM_A, 18DND-NAM_A,19RSW-NAM_ABC and 18DND-NAM_A, respectively (Fig. 1; Supplementary Table 4). For all traits, the performance was low in 20MRA-NAM_BC compared to the other environments. The performance of the check varieties in response to different environments followed the same pattern as the performance of the NAM RILs (Fig. 1).

The differences in the environments were further highlighted by the percentage of variation accounted for (%VAF) by the two factors of the FALMM we used to analyse the MET data. For GY, the %VAF factor_1 for the additive effects ranged from 14.6 to 99.9% meaning that it did not consistently explain the greater amount of variation in all sites. This indicates the presence of GEI. For TGW, the first factor accounted for more than 76% of the additive variance for all the sites indicating low GEI. For SCG, the first factor for additive effects accounted for at least 60% of the variance for all sites except for 17RSW_NAM_A and 18RSW-NAM_A while for HW at least 65% of the variance was explained by the first factor in all sites except for 19DND-NAM_C,19RSW-NAM_ABC and 20MRA-NAM_BC. The FA2 model generally showed a good fit for all the traits in most sites as evidenced by the high total %VAF the additive effects (Table 3).

**Table 3:** Percentage variation accounted from the FA2 model for additive effects in GY, TGW, SCG and HW in each of the nine environments

Trait	Trial	Additive genetic effects		
		%vaf factor_1	%vaf factor_2	Total %vaf
Yield	17RSW-NAM_A	65.1	0.232	65.4
	18DND-NAM_A	52.3	31.7	84.0
	18RSW-NAM_A	91.8	8.22	100
	18RSW-NAM_B	99.954	0.046	100
	19DND-NAM_B	80.2	19.8	100
	19DND-NAM_C	52.8	0.049	52.8
	19LKH-NAM_A	26.0	28.8	54.8
	19RSW-NAM_ABC	50.2	16.7	66.8
	20MRA-NAM_BC	14.6	85.4	100
TGW	17RSW-NAM_A	89.5	10.5	100
	18DND-NAM_A	86.6	5.22	91.8
	18RSW-NAM_A	97.6	2.36	100
	18RSW-NAM_B	88.2	2.73	91.0
	19DND-NAM_B	94.9	0.087	95.0
	19DND-NAM_C	91.2	8.81	100
	19LKH-NAM_A	93.4	6.65	100
	19RSW-NAM_ABC	98.1	1.94	100
	20MRA-NAM_BC	76.5	1.28	77.8
Screenings	17RSW-NAM_A	22.7	43.84	66.5
	18DND-NAM_A	96.8	3.23	100
	18RSW-NAM_A	40.3	59.7	100
	18RSW-NAM_B	89.2	0.198	89.4
	19DND-NAM_B	87.0	13.0	100
	19DND-NAM_C	62.4	37.6	100
	19LKH-NAM_A	77.6	4.82	82.5
	19RSW-NAM_ABC	97.5	2.50	100
	20MRA-NAM_BC	64.3	0.555	64.9
HW	17RSW-NAM_A	68.0	22.4	90.5
	18DND-NAM_A	77.9	0.119	78.1
	18RSW-NAM_A	77.2	5.14	82.4
	19DND-NAM_B	85.6	14.4	100
	19DND-NAM_C	34.6	65.4	100
	19LKH-NAM_A	66.1	33.9	100
	19RSW-NAM_ABC	51.9	8.96	60.9
	20MRA-NAM_BC	39.4	9.31	48.7

### Genetic correlations (***r***_g_) between paired environments

**Fig. 2:**
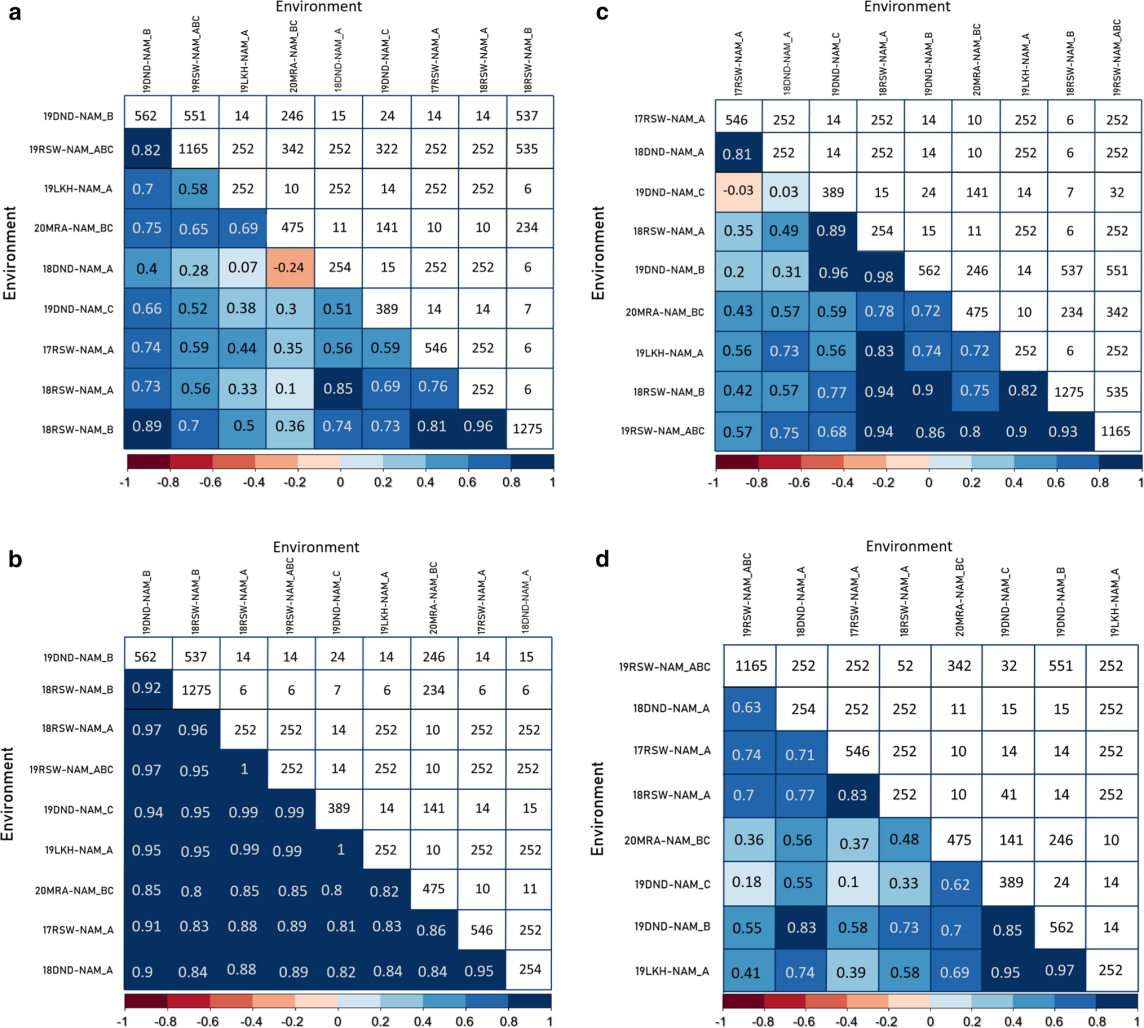
Heatmaps of the genetic correlations (*r*_g_) between pairs of environments estimated from variance–covariance of the FA2 model (lower triangle) and number of varieties in common between a pair of environments (upper triangle) for **a** GY **b** TGW **c** SCG **d** HW. Colours for the lower triangle range from dark blue for high positive *r*_g_ to dark red for strong negative *r*_g_*.* High *r*_g_ means low GEI

The genetic correlations (*r*_g_) between pairs of environments give a measure of the level of GEI between paired environments. Higher *r*_g_, indicates similar performance of genotypes between paired environments and hence low GEI. *r*_g_ between paired environments showed different patterns for different traits (Fig. 2). For GY (Fig. 2a), the *r*_g_ between environments ranged from − 0.24 to 0.96. 20MRA-NAM_BC had low correlation with the other sites except 19DND-NAM_B, 19RSW-NAM_ABC and 19LKH-NAM_A. 18DND-NAM_A also had low correlation (*r*_g_ < 0.6) with all the trials except 18RSW-NAM_A and 18RSW-NAM_B. Trial 19DND-NAM had high correlation (*r*_g_ ≥ 0.66) with all the trials except 18DND-NAM_A. The Roseworthy trials were generally highly correlated (≥ 0.7) with each other. 18RSW-NAM_A and 18DND-NAM_A were also highly correlated. For TGW (Fig. 2b), all the trials were highly correlated with each other, the *r*_g_ ranged from 0.8 to 0.99. For SCG (Fig. 2c), *r*_g_ ranged from − 0.03 to 0.98. Trial 17RSW-NAM_A had low correlation (*r*_g_ ≤ 0.57) with all the trials except 18RSW-NAM_A. 19RSW-NAM_ABC had high correlation (*r*_g_ ≥ 0.68) with all trials except 17RSW-NAM_A. For HW (Fig. 2d), the *r*_g_ ranged from 0.1 to 0.97. 20MRA-NAM_BC and 19DND-NAM_C had low correlations (*r*_g_ ≤ 0.56) with the other trials except with each other and 19DND-NAM_B and 19LKH-NAM_A.

### Detection of marker-trait associations

**Fig. 3:**
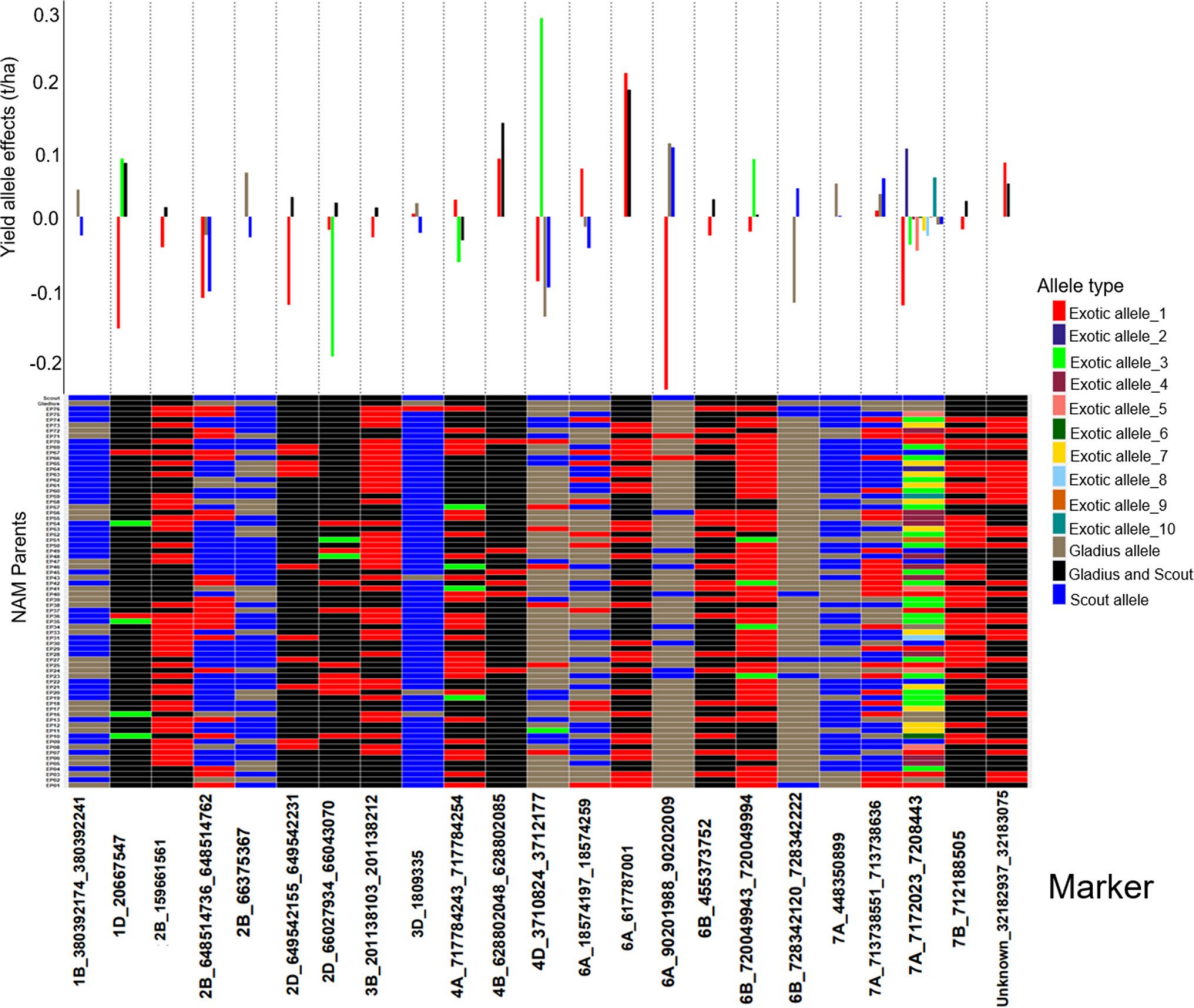
Distribution of allele effects (upper bar graph) of the detected MTA in the NAM RILs and NAM parents allele matrix showing allelic diversity in the parents for the detected MTA (lower heatmap) for GY. For the distribution of the allele effects, each bar represents an allele, the length of the bar denotes the size of the allele effect, and each column corresponds to the MTA detected by GWAS for GY. Collectively, the number of bars in each column of the bar graph corresponds to the number of alleles for the respective MTA. For the allele matrix, each row corresponds to the NAM parent, and each column corresponds to the MTA detected by GWAS for GY and each cell of the heatmap denotes an allele. Allele type refers to the source of the allele; exotic allele if the allele is only found in the exotic parents and is contributed to the NAM RILs by the exotic parent(s); Gladius allele if the allele in the exotic parent(s) and the NAM RILs is the same as the allele for Gladius recurrent parent; Gladius and Scout allele if both the recurrent parents share a similar allele and the exotic parent and NAM RILs also share the similar allele as Gladius and Scout; Scout allele if the allele in the exotic parent(s) and the NAM RILs is the same as the allele for Scout recurrent parent. The exotic alleles are numbered for each MTA and denote the amount of different exotic alleles per MTA

**Fig. 4:**
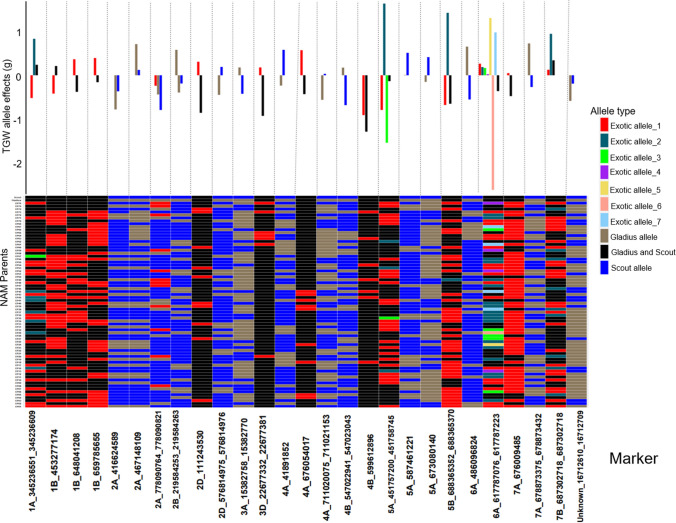
Distribution of allele effects (upper bar graph) of the detected MTA in the NAM RILs and NAM parents allele matrix showing allelic diversity in the parents for the detected MTA (lower heatmap) for TGW. For the distribution of the allele effects, each bar represents an allele, and the length of the bar denotes the size of the allele effect, and each column corresponds to the MTA detected by GWAS for TGW. Collectively, the number of bars in each column of the bar graph corresponds to the number of alleles for the respective MTA. For the allele matrix, each row corresponds to the NAM parent, each column corresponds to the MTA detected by GWAS for TGW and each cell of the heatmap denotes an allele. Allele type refers to the source of the allele; exotic allele if the allele is only found in the exotic parents and is contributed to the NAM RILs by the exotic parent(s); Gladius allele if the allele in the exotic parent(s) and the NAM RILs is the same as the allele for Gladius recurrent parent; Gladius and Scout allele if both the recurrent parents share a similar allele and the exotic parent and NAM RILs also share the similar allele as Gladius and Scout; Scout allele if the allele in the exotic parent(s) and the NAM RILs is the same as the allele for Scout recurrent parent. The exotic alleles are numbered for each MTA and denotes the amount of different exotic alleles per MTA

**Fig. 5:**
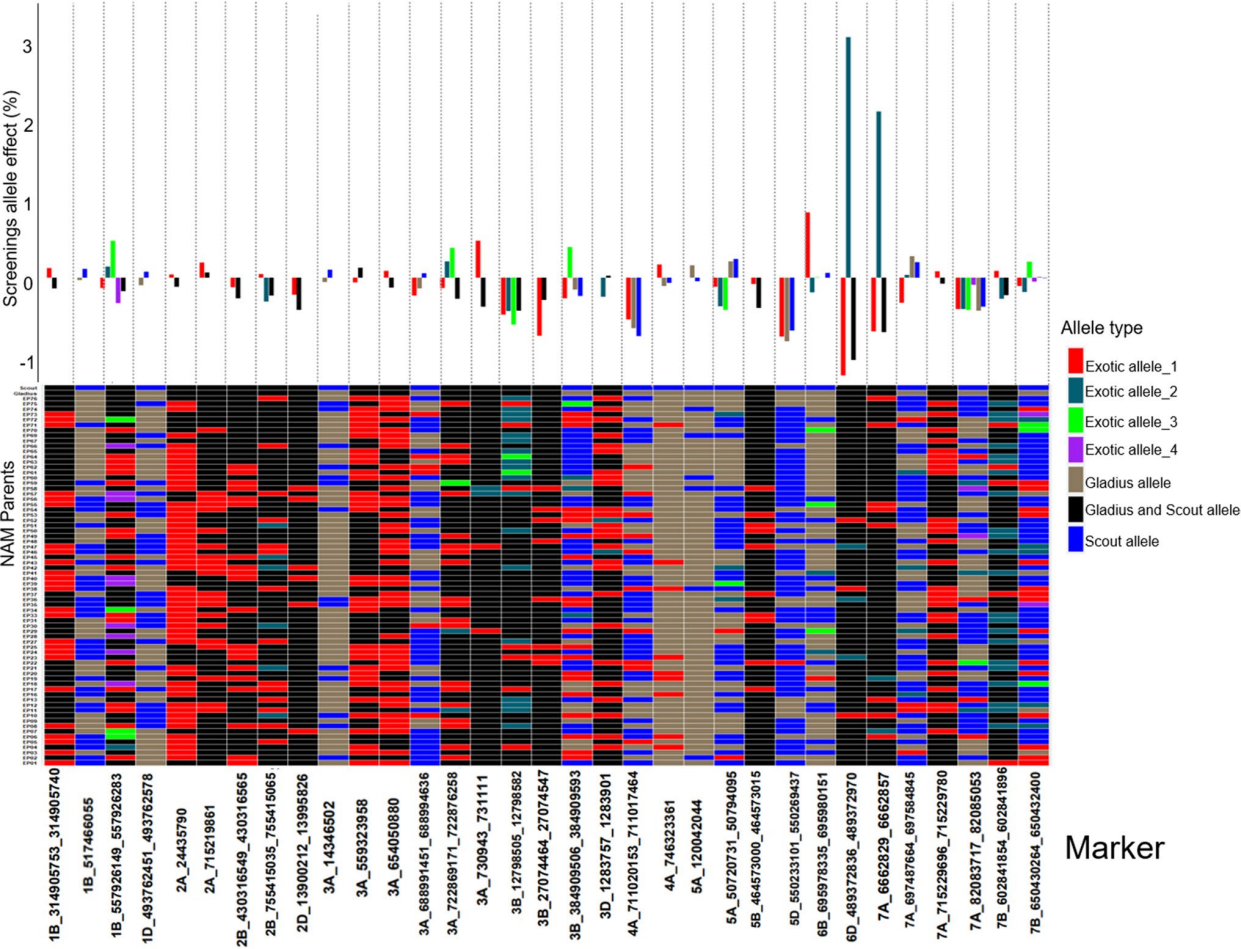
Distribution of allele effects (upper bar graph) of the detected MTA in the NAM RILs and NAM parents allele matrix showing allelic diversity in the parents for the detected MTA (lower heatmap) for SCG. For the distribution of the allele effects, each bar represents an allele and the length of the bar denotes the size of the allele effect and each column corresponds to the MTA detected by GWAS for SCG. Collectively, the number of bars in each column of the bar graph corresponds to the number of alleles for the respective MTA. For the allele matrix, each row corresponds to the NAM parent, each column corresponds to the MTA detected by GWAS for SCG, and each cell of the heatmap denotes an allele. Allele type refers to the source of the allele; exotic allele if the allele is only found in the exotic parents and is contributed to the NAM RILs by the exotic parent(s); Gladius allele if the allele in the exotic parent(s) and the NAM RILs is the same as the allele for Gladius recurrent parent; Gladius and Scout allele if both the recurrent parents share a similar allele and the exotic parent and NAM RILs also share the similar allele as Gladius and Scout; Scout allele if the allele in the exotic parent(s) and the NAM RILs is the same as the allele for Scout recurrent parent. The exotic alleles are numbered for each MTA and denote the amount of different exotic alleles per MTA

**Fig. 6:**
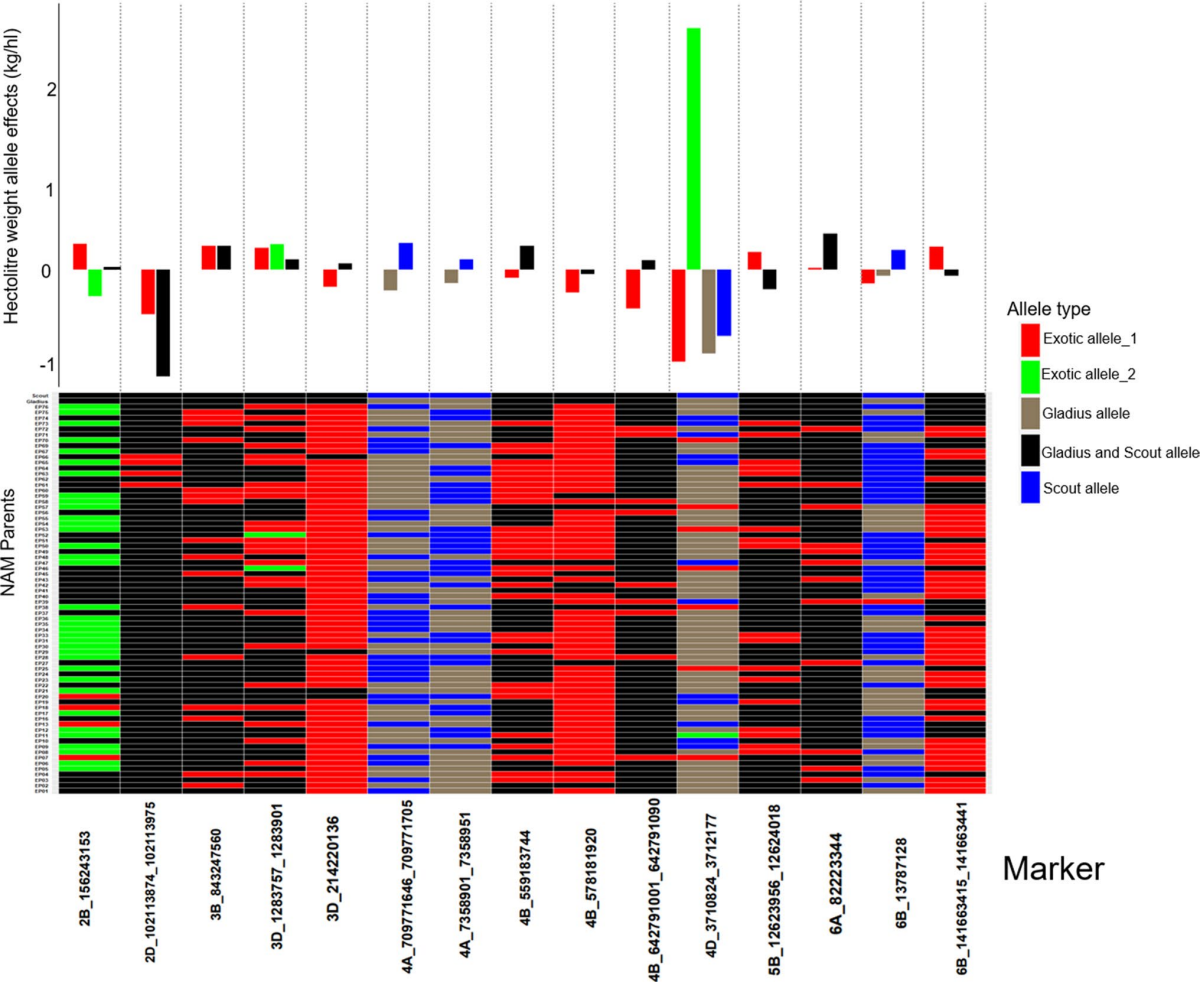
Distribution of allele effects (upper bar graph) of the detected MTA in the NAM RILs and NAM parents allele matrix showing allelic diversity in the parents for the detected MTA (lower heatmap) for HW. For the distribution of the allele effects, each bar represents an allele and the length of the bar denotes the size of the allele effect and each column corresponds to the MTA detected by GWAS for HW. Collectively, the number of bars in each column of the bar graph corresponds to the number of alleles for the respective MTA. For the allele matrix, each row corresponds to the NAM parent, each column corresponds to the MTA detected by GWAS for HW and each cell of the heatmap denotes an allele. Allele type refers to the source of the allele; exotic allele if the allele is only found in the exotic parents and is contributed to the NAM RILs by the exotic parent(s); Gladius allele if the allele in the exotic parent(s) and the NAM RILs is the same as the allele for Gladius recurrent parent; Gladius and Scout allele if both the recurrent parents share a similar allele and the exotic parent and NAM RILs also share the similar allele as Gladius and Scout; Scout allele if the allele in the exotic parent(s) and the NAM RILs is the same as the allele for Scout recurrent parent. The exotic alleles are numbered for each MTA and denote the amount of different exotic alleles per MTA

In this study, we used a GWAS approach to map marker-trait associations (MTAs) for GY, TGW, HW and SCG in the NAM population. We detected a total of 98 significant MTAs for the four traits (Tables 4, 5, 6, 7). Seventy-four MTAs had alleles that were derived only from the exotic parents and were not found in either of the two recurrent parents (Figs. 3, 4, 5, 6). The exotic alleles had favourable effects at 46 of the 74 MTAs (Figs. 3, 4, 5, 6). One MTA was common between GY and HW, and another MTA was common between HW and SCG. SCG (Table 6) had the highest number (33) of MTAs detected, while HW (Table 8) had the least (15). The exotic parents showed diversity in the detected MTA for each trait. Each of the MTAs detected for HW had a genetic contribution (*R*^2^) greater than 2%. In the following, we present the QTL for each trait separately.

**Table 4:** Detected MTA for GY across multiple environments and the related chromosome (Chr), position in base pairs (bp), number of alleles, significance level (*p* value) and the genetic contribution (*R*^2^%)

Marker	Chr	Position (bp)	Number of alleles	*p* value	*R*^2^%
2B_66375367^a^	2B	66,375,367	2	1.92E−17	6.09
7A_7172023_7208443	7A	7,172,023–7,208,443	12	2.02E−06	4.77
6B_728342120_728342222	6B	728,342,120–728,342,222	2	3.28E−09	3.02
2B_648514736_648514762	2B	648,514,736 648,514,762	3	1.13E−06	2.67
4B_628802048_628802085	4B	628,802,048–628,802,085	2	2.85E−08	2.64
6A_18574197_18574259	6A	18,574,197–18,574,259	3	4.17E−06	2.32
1B_380392174_380392241	1B	380,392,174–380,392,241	2	1.22E−06	2.02
6A_617787001	6A	617,787,001	2	6.79E−06	1.72
2D_66027934_66043070	2D	66,027,934–66,043,070	3	9.28E−05	1.67
U_32182937_32183075	Unassigned	32,182,937–32,183,075	2	9.24E−06	1.66
4A_717784243_717784254	4A	717,784,243–717,784,254	3	1.57E−04	1.60
7B_712188505	7B	712,188,505	2	1.33E−05	1.57
7A_713738551_713738636	7A	713,738,551–713,738,636	3	3.19E−04	1.57
1D_20667547	1D	20,667,547	2	7.82E−05	1.37
4D_3710824_3712177^b^	4D	3,710,824–3,712,177	4	6.83E−04	1.35
6B_720049944_720049993	6B	720,049,944–720,049,993	3	3.10E−04	1.27
7A_448350899^c^	7A	448,350,899	2	1.58E−04	1.27
2D_649542155_649542231	2D	649,542,155–649,542,231	2	1.36E−04	1.23
6A_90201988_90202009	6A	90,201,988–90,202,009	4	6.05E−04	1.16
2B_159661561	2B	159,661,561	2	2.48E−04	1.06
3D_1809335	3D	1,809,335	2	6.75E−04	1.01
3B_201138103_201138212	3B	201,138,103–201,138,212	2	8.39E−04	0.98
6B_455373752	6B	455,373,752	2	6.33E−04	0.97

Positions are based on the IWGSC genome assembly of Chinese Spring version 2.0. For markers in a linkage disequilibrium block the position is given as a range. QTL are sorted by *R*^2^%

^a^MTA located 3 Mb from the *Ppd-B1* locus

^b^MTA collocated with HW MTA

^c^MTA collocated with a cluster of yield QTL reported by (Quarrie et al. 2006)

**Table 5:** Detected MTA for TGW across multiple environments and the related chromosome (Chr), position in base pairs (bp), number of alleles, significance level (*p* value) and the genetic contribution (*R*^2^%)

Marker	Chr	Position (bp)	Number of alleles	*p* value	*R*^2^%
1A_345236551_345236609	1A	345,236,551–345,236,609	3	1.42E−14	3.9
1B_453277174	1B	453,277,174	2	5.79E−14	3.6
1B_648041208	1B	648,041,208	2	2.16E−11	2.8
1B_659785655	1B	659,785,655	2	26.34E−11	2.7
2A_416624589	2A	416,624,589	2	22.18E−10	2.5
2A_467148109	2A	467,148,109	2	4.92E−09	2.3
4B_547022941_547023043	4B	547,022,941–547,023,043	2	5.70E−05	1.9
2A_778090764_778090821	2A	778,090,764–778,090,821	3	1.00E−07	1.7
2B_219584253_219584263	2B	219,584,253–219,584,263	3	4.58E−07	1.5
2D_576814975_576814976	2D	576,814,975–576,814,976	2	5.36E−07	1.5
2D_111243530	2D	111,243,530	2	7.15E−07	1.5
4A_676054017	4A	676,054,017	2	4.00E−05	1.4
7A_678873375_678873432	7A	678,873,375–678,873,432	2	2.04E−04	1.2
5A_451757200_451758745	5A	451,757,200–451,758,745	4	6.85E−05	1.2
4A_711020075_711021153	4A	711,020,075–711,021,153	2	4.02E−05	1.1
4A_41891852	4A	41,891,852	2	1.70E−05	1.1
3A_15382758_15382770	3A	15,382,758–15,382,770	2	1.08E−05	1.1
3D_22677332_22677381	3D	22,677,332–22,677,381	2	2.56E−05	1.0
4B_599612896	4B	599,612,896	2	6.54E−05	1.0
6A_486096824	6A	486,096,824	2	2.79E−04	1.0
5B_688365352_688365370	5B	688,365,352–688,365,370	3	3.10E−04	0.9
5A_587461221^a^	5A	587,461,221	2	2.32E−04	0.8
6A_617787076_617787223	6A	617,787,076–617,787,223	8	3.67E−04	0.8
7A_676009485	7A	676,009,485	2	2.25E−04	0.8
5A_673080140	5A	673,080,140	2	2.82E−04	0.8
7B_687302718_687302718	7B	687,302,718–687,302,718	3	8.62E−04	0.7
Unknown_16712610_16712709		16,712,610–16,712,709	2	5.14E−04	0.7

Positions are based on the IWGSC genome assembly of Chinese Spring version 2.0. For markers in a linkage disequilibrium block the position is given as a range. QTL are sorted by *R*^2^%

^a^ MTA located about 1,7 Mb from the *VRN−A1* locus

**Table 6:** Detected MTA for SCG across multiple environments and the related chromosome (Chr), position in base pairs (bp), number of alleles, significance level (*p* value) and the genetic contribution (*R*^2^%)

Marker	Chr	Position (bp)	Number of alleles	*p* value	*R*^2^%
4A_711017464_711020153	4A	711,017,464–711,020,153	3	5.02E−10	3.0
5D_550233101_550269437	5D	550,233,101–550,269,437	3	1.15E−07	2.8
1D_493762451_493762578	1D	493,762,451–493,762,578	2	1.12E−08	2.4
5A_50720731_50794095	5A	50,720,731–50,794,095	5	9.18E−08	1.9
6D_489372836_489372970	6D	489,372,836–489,372,970	3	3.56E−08	1.9
2B_755415035_755415065	2B	755,415,035–755,415,065	3	3.10E−07	1.9
3A_688991451_688994636	3A	688,991,451–688,994,636	3	2.66E−05	1.8
7B_602841854_602841896	7B	602,841,854–602,841,896	3	2.09E−05	1.7
1B_314905740_314905753	1B	314,905,740–314,905,753	2	1.37E−06	1.7
3A_730943_731111	3A	7309,43–731,111	2	2.24E−06	1.6
3B_384909506_384909593	3B	384,909,506–384,909,593	4	3.15E−04	1.6
6B_695978335_695980151	6B	695,978,335–695,980,151	5	2.82E−05	1.5
2D_13900212_13995826	2D	13,900,212–13,995,826	2	2.50E−05	1.3
3D_1283757_1283901^a^	3D	1,283,757–1,283,901	3	3.12E−04	1.3
1B_557926149_557926283	1B	557,926,149–557,926,283	5	3.08E−05	1.2
3A_654050880	3A	654,050,880	2	9.07E−04	1.2
3A_559323958	3A	559,323,958	2	5.36E−04	1.1
4A_746323361	4A	746,323,361	3	1.08E−04	1.1
2B_430316549_430316565	2B	430,316,549–430,316,565	2	3.24E−05	1.1
5A_120042044	5A	120,042,044	2	5.16E−06	1.1
3B_27074464_27074547	3B	27,074,464–27,074,547	2	2.79E−05	1.0
3A_14346502	3A	14,346,502	2	6.62E−04	0.9
7A_6662829_6662857	7A	6,662,829–6,662,857	3	1.43E−04	0.9
7A_82083717_82085053	7A	82,083,717–82,085,053	6	6.99E−05	0.9
7B_650430264_650432400	7B	650,430,264–650,432,400	6	4.57E−04	0.9
3B_12798505_12798582	3B	12,798,505–12,798,582	4	2.94E−04	0.8
2A_24435790	2A	24,435,790	2	1.72E−04	0.8
7A_715229696_715229780	7A	715,229,696–715,229,780	2	2.23E−04	0.8
7A_697487664_697584845	7A	697,487,664–697,584,845	4	1.46E−04	0.8
1B_517466055	1B	517,466,055	2	4.99E−04	0.8
3A_722869171_722876258	3A	722,869,171–722,876,258	4	8.33E−04	0.7
2A_715219861	2A	715,219,861	2	4.60E−04	0.7
5B_464573000_464573015	5B	464,573,000–464,573,015	2	7.77E−04	0.6

Positions are based on the IWGSC genome assembly of Chinese Spring version 2.0. For markers in a linkage disequilibrium block the position is given as a range. QTL are sorted by *R*^2^%

^a^ MTA collocated with HW

**Table 7:** Location of MTA which coincide with previously reported QTL/genes from other studies

Marker	Chr:Pos	Trait	Closest previously reported QTL/Gene
2B_66375367	2B: 66,375,367	GY	*Ppd-B1* (Scarth and Law 1984)
7A_448350899	7A: 448,350,899	GY	SSR locus *Xbarc108* (Quarrie et al. 2006)
5A_587461221	5A: 587,461,221	TGW	*VRN-A1* (Trevaskis et al. 2003)
4A_717784243_717784254	4A: 717,784,243 −717,784,254	GY	*wPt-3150* (Maphosa et al. 2014)
3D_1809335	3D: 1,809,335	GY	*wPt-7984* (Maphosa et al. 2014)
2D_13900212_13995826	2D:13,900,212–13,995,826	SCG	SSR locus *cfd36 (*Maphosa et al. 2014)
1A_345236551_345236609	1A:345,236,551–345,236,609	TGW	*Qtgw.caas-1AL (*Yang et al. 2020*)*
1B_648041208	1B: 648,041,208	TGW	*QYld.aww-1B.2* (Tura et al. 2020)

Positions are based on the IWGSC genome assembly of Chinese Spring version 2.0. For markers in a linkage disequilibrium block, the position is given as a range

### GY

We identified 23 MTAs for GY which were spread across 13 of the wheat's chromosomes (Table 4). The most significant of these had -log P significance level of 16.7 on chromosome 2B. The genetic contribution (*R*^2^%) of each MTA ranged from 1.0 to 6.1% with a total of 44.9%. (Table 4). The chromosome 2B MTA had the greatest genetic contribution of 6.09% and was located 3 Mb from the *Ppd-B1* locus. One MTA on chromosome 7A had a genetic contribution of 1.27% and was found to be collocated with a cluster of yield QTL that was detected in a study by Quarrie et al. (2006).

The number of alleles per MTA ranged from 2 to 12 with a total of 67 alleles (Table 4; Fig. 3). Thirty-three alleles were related to positive increases in GY of up to 284 kg/ha, and 34 alleles were related to GY reductions of up to 247 kg/ha. Together, exotic parents contributed 33 alleles that were distinct from the recurrent parents’ alleles. These exotic alleles were present at 19 MTAs and included the most favourable allele related to an increase of 284 kg/ha in GY and the least favourable allele that reduced GY by 247 kg/ha. The exotic alleles had favourable effects at eight QTL (Fig. 3). The exotic parents showed diversity in the 23 MTAs particularly on one MTA located on chromosome 7A where up to ten different exotic alleles were detected. Some exotic alleles were rare, being found in only one exotic parent (Fig. 3).

### TGW

For TGW, we identified 27 MTAs with a - log *P* significance level ranging from 3.2 to 13.8. Twenty-six MTAs mapped to 14 chromosomes and 1 MTA mapped to an unassigned region of the genome (Table 5). The genetic contribution (*R*^2^%) per MTA ranged from 0.7 to 3.9% with a sum total of 41.3% of the phenotypic variation (Table 5). The number of alleles per MTA ranged from 2 to 8 alleles with a total of 67 alleles with both positive and negative allele effects. Thirty-three alleles were associated with TGW increases of up to 1.6 g, while 34 alleles were associated with TGW decreases of up to 2.6 g (Fig. 4). Twenty-five alleles with effects ranging from − 2.64 g to 1.65 g were unique to the exotic parents and were present in 14 MTAs (Fig. 4). The exotic allele had favourable effects on TGW at 11 MTAs. Figure 4 shows the allele effect distribution and the allele constitution of the NAM parents for the 27 MTAs. The exotic parents showed diversity in the MTA. The MTA on chromosome 6A was the most diverse with seven different exotic alleles. A QTL located on chromosome 5A was about 1.7 Mb from the vernalisation gene (*VRN-A1*). Two more loci were also located close to QTL detected in previous studies (Table 5).

### SCG

We identified 33 MTAs for SCG which mapped to 16 chromosomes with a -log P significance level ranging from 3.1 to 9.3 (Table 6). Each MTA had a genetic contribution (*R*^2^%) ranging between 0.6% and 2.9%, with a sum total of 43.5% of the phenotypic variation (Table 6). In total, there were 100 alleles for the 33 MTAs and the number of alleles per MTA ranged from 2 to 6. Figure 5 shows the distribution of allele effects and the allelic diversity of the NAM parents for SCG. The estimated allele effects ranged from − -1.2 to 3%. Sixty-four alleles reduced SCG, while 36 alleles increased SCG. Fifty-three out of the 100 alleles were unique to the exotic parents and were present at 28 MTAs with estimated effects ranging from − 1.2 to 3% (Fig. 5). The favourable effects of the exotic alleles were present on 22 MTAs. Three MTAs had four different exotic alleles.

### HW

We identified 15 MTAs for HW which mapped to chromosomes 2B, 2D, 3B, 3D (two MTAs), 4A (two MTAs), 4B (three MTAs), 4D, 5B, 6A and 6B (two MTAs) with a  −log P significance level ranging from 3.0 to 9.9 (Table 8). One MTA on chromosome 3D co-located with a QTL for SCG, and another MTA on chromosome 4D co-located with an MTA for GY. Each MTA had a genetic contribution (*R*^2^%) ranging between 2.4 and 9.3%, with a sum total of 58.1% of the phenotypic variation (Table 8). Figure 6 shows the distribution of allele effects and the allelic diversity of the NAM parents for HW. There was a total of 35 alleles for the 15 MTAs, and the number of alleles per MTA ranged from 2 to 4. The favourable alleles were associated with HW increases of up to 2.5 kg/hL, and the unfavourable alleles were associated with a decrease of up to 1.1 kg/hL in HW. The exotic NAM parents contributed sixteen alleles which were present at 13 MTAs (Fig. 6). One of the exotic alleles was associated with the largest increase in HW. The exotic parents showed diversity in MTA, but the MTA for HW was not as diverse as some of the MTA detected for the other three traits.

## Discussion

As global climate changes, the severity and frequency of drought and heat stress on crop production are expected to increase. Drought and heat stress are the major abiotic stresses limiting wheat production globally. Drought is when a plant experiences water stress at levels that are sufficient to affect plant growth rates (Lobell et al. 2015). Heat stress is when temperature rises beyond a threshold level for a period of time sufficient to cause irreversible damage to plant growth and development (Wahid et al. 2007). Wheat is very sensitive to heat stress and the effect of heat stress depends on the timing (wheat growth stage during heat stress) and length of exposure to heat stress (Akter and Rafiqul Islam 2017). Under heat stress conditions, wheat yields are reduced due to a reduction in the duration of the flowering and grain filling stages (Kamrun et al. 2010). Heat stress occurring at the flowering stage usually reduces the number of grains, while heat stress at the grain filling stage reduces the grain weight (Kamrun et al. 2010). In Australia, drought and heat are regular climatic features, and their impact on wheat yield is more pronounced when drought coincides with heat waves above 32 °C during heading and grain filling stages. In a bad year, drought can reduce wheat yields in Australia by 50% (Roy et al. 2021). In 2006, wheat yields decreased by 46% from the long-term mean due to drought (FAO 2013). With an average annual production worth $7.1 billion (GRDC 2018), drought can cost the Australian economy around $3.2 billion. Australian wheat is grown in the wheat belt that extends from the southwest of Western Australia, through South Australia, Victoria, New South Wales and into Southern Queensland (Zeleke 2021) and is mostly produced under rainfed/dryland conditions which makes it more prone to drought and heat stresses. Furthermore, the Australian wheat growing environments are highly variable mainly due to fluctuations in rainfall over years and regions. Differences in soil type, day length and sowing time over regions also contribute to the variability of the Australian wheat growing regions. In the present study, wheat was grown and evaluated in nine trials grown across the Australian wheat belt over three years.

The variability of environments was evident in this study as both the NAM RILs and check varieties performed differently in each environment. For the NAM set A trials, for example, average GY was higher in the Roseworthy 17RSW-NAM_A trial followed by the Dandaragan 18DND-NAM_A, the Roseworthy 18RSW-NAM_A and the Lockhart 19LKH-NAM_A trials, respectively (Fig. 1). The check varieties also followed the same trend in these trials. In general, the Roseworthy 2017 and Dandaragan 2018 trials experienced a good season with above-average rainfall, while in 2019, the Lockhart trial experienced drought and heat stress (Supplementary Table 1). While the performance differences of the NAM RILs can be attributed to differences in weather data (rainfall and temperature), differences in soil type, day length and sowing times might also have contributed to the differences in the performance of NAM RILs and check varieties in these trials.

The FA2 model we used to analyse the MET data confirmed the presence of substantial *G* × *E* interaction of the additive effects particularly for GY and to a lesser extend SCG and HW. This is apparent with the proportions (%VAF) of additive genetic variance explained by the two factors individually and in combination (Table 3) and the genetic correlation heatmaps (Fig. 2 a, c, d) for these traits. For GY, Table 3 shows heterogeneity in the %VAF between sites for the two factors. GY is a complex trait whose expression is influenced by the environment. Hence, the presence of GEI for GY is expected. For TGW, factor_1 (Table 3) of the FA2 model explains most of the variation for all the environments and, in combination, both factors explain close to 100% variation for all the sites. The genetic correlation (Fig. 2b) between pairs of environments was also greater than 0.8 between all the environments. This shows lack of GEI for this trait. The nature of the lack of GEI for TGW and the stability of TGW across different environments and years can be attributed to the fact that TGW is under strong genetic control (Zanke et al. 2015). High heritability estimates and major stable QTL have been reported for TGW (Schierenbeck et al. 2021; Yang et al. 2020).

In plant breeding, the interaction between the environment and the genotype poses a challenge in the development of improved varieties especially when there is a significant change in the ranking of genotypes across environments (Cooper and DeLacy 1994). Phenotypic evaluation of genetic material for important traits in METs provides a way of effectively measuring G × E and identifying stable genotypes and environments suited for specific genotypes (Elias et al. 2016; Smith et al. 2021). In this study, we also identified NAM RILs SCEP20-006 and SCEP43-005 (Fig. 1a; Supplementary Table 3) which showed stable GY performance across multiple environments. These RILs show that besides enhancing QTL mapping, NAM populations can provide germplasm that can be incorporated into wheat breeding programmes.

### Detection of MTA through GWAS

Wheat is adapted to diverse geographical regions of the world because of its genetic potential to synchronise its flowering time with favourable environmental conditions (Kamran et al. 2014). This photoperiod response mechanism is crucial for maximising GY and is partly controlled by the *Ppd-A1*, *Ppd-B1*, and *Ppd-D1* genes located on the short arms of chromosomes 2A, 2B and 2D, respectively (Scarth and Law 1984). We detected an MTA on chromosome 2B (Table 4) which we speculate might be associated with the *Ppd-B1* locus considering it explains the highest amount of the phenotypic variation and is only about 3 Mb from *Ppd-B1*. Quarrie et al. (2005) and Quarrie et al. (2006) reported the presence of yield QTL on chromosomes 7A and 7B. Similarly, in this study, we detected three MTAs for GY on chromosome 7A (Table 4), one of which is in close proximity with a cluster of highly significant yield QTL reported by Quarrie et al. (2006). In a Drysdale × Gladius RIL population, Maphosa et al. (2014) detected a GY and two SCG QTL with *cfd36*, *wPt-7984* and *wPt-3150* as their closest markers, respectively. We detected three MTAs for GY, SCG and HW (Tables 4, 6, 8) which are also close to these markers based on the markers’ IWGSC RefSeq v2.1 (Zhu et al. 2021) genome positions (Blake et al. 2019). Maphosa et al. (2014), reported marker cfd36 to be on chromosome 2A, and however, a search in the GrainGenes database (Blake et al. 2019) shows its location to be on chromosome 2D about 661 Kb from our SCG MTA on chromosome 2D. Likewise, the *wPt-7984* marker was reported to be on chromosome 3B, but its position according to the GrainGenes database is on chromosome 3D about 1 Mb from our 3D MTA that is common between SCG and HW (Tables 6, 8). Since the population used by Maphosa et al. (2014) shares a common parent, Gladius, with the OzNAM, it is possible that these MTAs are the same. Gladius has a favourable allele at these loci, and however, in some instances, the allele is not the most beneficial. It is also possible that the MTA we detected and MTA detected by Maphosa et al. (2014) are homeologs.

**Table 8:** Detected MTA for HW across multiple environments and the related chromosome (Chr), position in base pairs (bp), number of alleles, significance level (*p* value) and the genetic contribution (*R*^2^%). For markers in a linkage disequilibrium block, the position is given as a range

Marker	Chr	Position (bp)	Number of alleles	*p* value	*R*^2^%
4A_709771646_709771705	4A	709,771,646–709,771,705	2	1.22E−10	9.3
4B_559183744	4B	559,183,744	2	6.61E−08	6.6
2D_102113874_102113975	2D	102,113,874–102,113,975	2	2.25E−05	4.2
4B_642791001_642791090	4B	642,791,001–642,791,090	2	2.22E−04	4.1
6B_141663415_141663441	6B	141,663,415–141,663,441	2	2.12E−04	4.1
4D_3710824_3712177^a^	4D	3,710,824–3,712,177	4	2.76E−05	4.1
3D_1283757_1283901^b^	3D	1,283,757–1,283,901	3	1.21E−04	3.3
3D_214220136	3D	214,220,136	2	1.33E−04	3.3
6B_13787128	6B	13,787,128	3	1.92E−04	3.3
3B_843247560	3B	843,247,560	2	2.97E−04	2.9
5B_12623956_12624018	5B	12,623,956–12,624,018	2	3.58E−04	2.9
4B_578181920	4B	578,181,920	2	5.65E−04	2.6
6A_82223344	6A	82,223,344	2	6.81E−04	2.6
2B_156243153	2B	156,243,153	3	8.29E−04	2.5
4A_7358901_7358951	4A	7,358,901–7,358,951	2	9.27E−04	2.4

Positions are based on the IWGSC genome assembly of Chinese Spring version 2.0. For markers in a linkage disequilibrium block, the position is given as a range. QTL are sorted by *R*^2^%

^a^MTA collocated with GY MTA

^b^MTA collocated with SCG MTA

The *VRN-A1* loci in wheat influence floral activation and consequently GY (Trevaskis et al. 2003). We detected an MTA for TGW located close to the *VRN-A1* locus on chromosome 5A (Table 5). Since TGW is a major component of GY, it is possible that the *VRN-A1* loci also influence TGW. We also found a QTL on chromosome 1A that was within the detected interval of a TGW QTL (Qtgw.caas-1AL) previously reported by Yang et al. (2020). Another QTL on chromosome 1B was close to a yield QTL (*QYld.aww-1B.2*) previously identified by Tura et al. (2020).

Many of the MTAs we detected in this study are potentially novel, since to the best of our knowledge, no other study has reported the presence of MTA at the same positions. QTL mapping for phenotypic data measured in multiple environments is usually done by analysing genotypic values averaged across environments or is performed separately for each environment (Garin et al. 2020). These methods ignore the genetic correlations between environments and are therefore prone to increased false positives in QTL detection (Piepho 2005). In general, using a correct variance–covariance structure for multi-environment data improves the detection of QTL (van Eeuwijk et al. 2010). Here, for our MET data, we used a one-stage analysis FA2 model that effectively models genetic variance across environments and genetic covariance between pairs of environments. The FA2 model coupled with the use of a multi-parent population enabled the detection of new MTA.

Bi-parental populations and diversity panels have been the commonly used types of mapping populations in wheat QTL mapping studies (Myles et al. 2009). While these populations have been successful in detecting significant QTL, they are limited compared to multi-parent NAM populations (Korte and Farlow 2013; Yu et al. 2008). NAM populations build upon the genetic principles of bi-parental populations and diversity panels and therefore have the advantages of having high allelic diversity, high power and resolution for QTL mapping while eliminating the confounding effect of population structure. NAM populations provide an opportunity to effectively capture diverse as well as rare alleles per locus from the founder lines unlike bi-parental populations (McMullen et al. 2009). Often a QTL has multiple alleles per locus and these can be easily detected in NAM populations. In this study, the NAM population has been valuable in dissecting the genetic architecture of grain yield and yield-related traits in wheat. Novel MTAs with multiple alleles were detected.

In summary, a total of 98 MTAs with multiple alleles associated with GY, TGW, SCG and HW were identified in this study. To the best of our knowledge, many of the MTAs we identified are novel and some of their most favourable alleles for each trait originated from the exotic parents. Two NAM RILs with superior performance in GY in most of the environments provided evidence of positive transgressive segregation in the NAM. The results from this study highlight the value of the NAM population in dissecting the genetic architecture of complex traits and provide germplasm for breeding programmes. Moreover, the study confirms the usefulness of exotic germplasm in introducing new and favourable genetic diversity in elite wheat gene pools.

## Supplementary Information

Below is the link to the electronic supplementary material.

Supplementary file1 (DOCX 11 kb)

Supplementary file2 (XLSX 2011 kb)

Supplementary file3 (DOCX 130 kb)

## Data Availability

The datasets generated during and/or analysed during the current study are available from the corresponding author on reasonable request.
