# Supplementary material for: Nested association mapping-based GWAS for grain yield and related traits in wheat grown under diverse Australian environments
Source: Theor Appl Genet. 2022 Oct 7;135(12):4437–56. doi: 10.1007/s00122-022-04230-9 (PMC9734238; doi:10.1007/s00122-022-04230-9)
Supplement: Supplementary file 1 — Supplementary file1 (DOCX 11 kb) [file 122_2022_4230_MOESM1_ESM.docx]

**Supplementary Information**

Supplementary Table 1. List of check varieties and the number of times they were replicated in each of the environments.

Supplementary Table 2. Trial description, design, duration and weather data for each of the trials in the multi-environmental trials.

Supplementary Table 3. Characterisation of the allele effects and allelic combinations at all the significant GY MTA loci in SCEP20-006 and SCEP 43-005.

Supplementary Table 4. Phenotypic performance (BLUPs) of the target traits (GY, TGW, SCG, HW) in each environment for each genotype.

Supplementary table 5. Allele and allele effects combinations at all significant GY MTA loci across all NAM RILs

Supplementary Fig. S1 Pearson correlation analysis of Grain Yield (GY), Thousand Grain Weight (TGW), Hectolitre Weight (HW) and Screenings (SCG)
