# Supplementary material for: Nested association mapping-based GWAS for grain yield and related traits in wheat grown under diverse Australian environments
Source: Theor Appl Genet. 2022 Oct 7;135(12):4437–56. doi: 10.1007/s00122-022-04230-9 (PMC9734238; doi:10.1007/s00122-022-04230-9)
Supplement: Supplementary file 3 — Supplementary file3 (DOCX 130 kb) [file 122_2022_4230_MOESM3_ESM.docx]

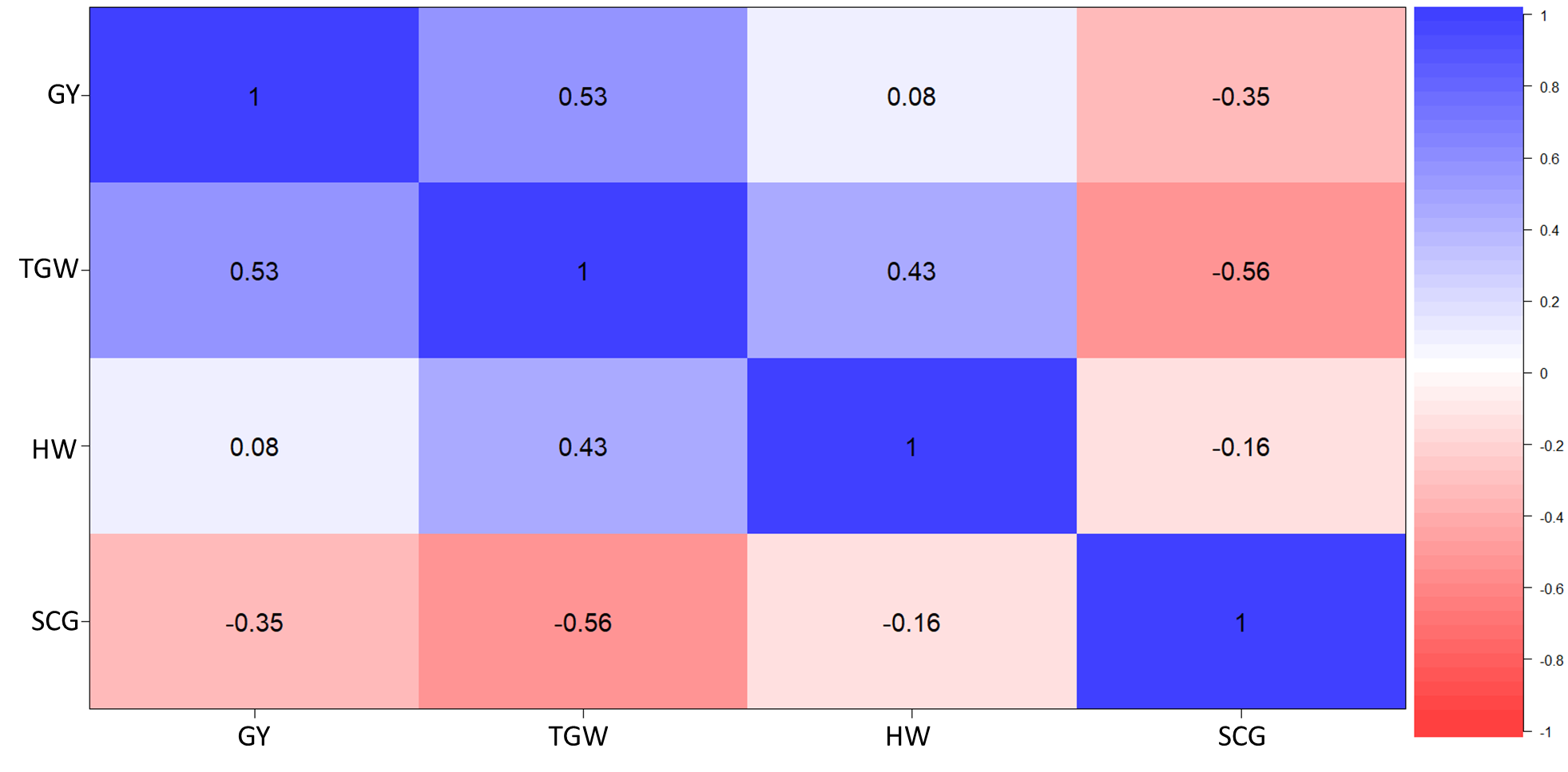


**Fig. S1** Pearson correlation analysis of Grain Yield (GY), Thousand Grain Weight (TGW), Hectolitre Weight (HW) and Screenings (SCG).
